# Digital pathways beyond Western-centric participants

**DOI:** 10.3758/s13428-025-02751-x

**Published:** 2025-07-18

**Authors:** Edmond Awad

**Affiliations:** 1 Department of Economics, University of Exeter, Exeter, UK; 2 Uehiro Oxford Institute, University of Oxford, Oxford, UK; 3 Center for Humans and Machines, Max Planck Institute for Human Development, Berlin, Germany

**Keywords:** Survey methodology, Cross-cultural research, Digital data collection, Online platforms, Cultural generalisability

## Abstract

In 2010, Henrich and colleagues published a seminal article in which they noted that (1) studies in social and behavioural sciences oversample from Western, educated, industrialised, rich, and democratic (WEIRD) individuals, and (2) WEIRD subjects are particularly unusual compared to the rest of the world population with respect to several factors. Despite the positive reception of this article, not much has changed in the years to follow. For instance, reviews of recent papers in leading psychology journals reveal that only a small proportion of the studied samples originate from non-Western countries. This sampling bias cannot be excused for lack of means. The digital age has opened several opportunities to facilitate and support social science research with subjects from non-WEIRD backgrounds. In this article, I provide an overview of such tools and comment on the advantages and disadvantages of each.

## Introduction

**Fig. 1:**
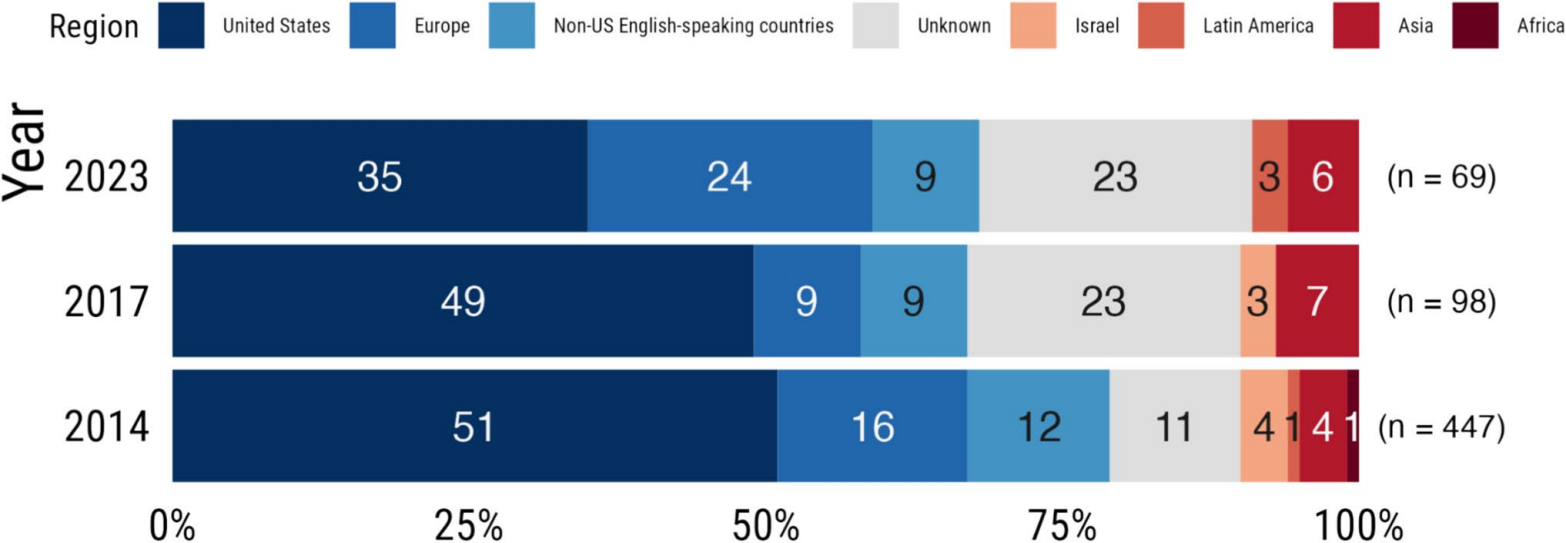
Percentage of the regional location of samples published in the *Psychological Science* journal in years 2014 and 2017 (first three issues) (Rad et al., 2018, Tables 1 and 2) and year 2023 (first three issues)

In the first three months of 2023, *Psychological Science* published 26 original research studies. Only 9% of the 69 samples of participants in these studies were from non-Western countries, including Asia, Africa, and Latin America (see Fig. 1). This shows no improvement from both 2014 and 2017, when 10% of the 447 and 98 participant samples, respectively, were from non-Western countries (Rad et al., 2018). And of the studies in 2014, 84% failed to suggest studying the phenomena in other cultures, implying generalisability beyond specific cultural contexts. This trend is not isolated to the *Psychological Science* journal. The same trend is echoed in six other psychology journals for the period of 2014 to 2018 (Thalmayer et al., 2021), top developmental psychology journals from 2006 to 2010 (Nielsen et al., 2017), and in more recent studies across various areas such as adolescent mental disorders (Erskine et al., 2017), environmental psychology (Tam & Milfont, 2020), sex and intimate relationship research (Klein et al., 2022; McGorray et al., 2023; Williamson et al., 2022), and human–computer interaction (HCI) (Linxen et al., 2021). It also extends to other social sciences from cognitive science to sociology, demography, political science, and behavioural economics. Oversampling participants from the Western world with the goal of generalising to all humans is worrying since there is established evidence that people in the Western world are particularly different from the rest (Henrich et al., 2010a).

**Table 1:** Overview of digital pathways

Approach	Strategic justification	Study design considerations	Operational setup	Quality assurance	Resource & risk profile
Targeted site selection, e.g., Ornstein, 1972)	Appropriate when theory suggests key dimensions of cultural variation, and resource constraints prevent broader coverage	Site selection should be based on theory or multidimensional frameworks such as WEIRD-scale (Muthukrishna et al., 2020) or the Inglehart–Welzel cultural map (Inglehart & Welzel, 2005). Avoid oversimplified East–West contrasts (Broesch et al., 2020; Masuda et al., 2020)	Identify 2–5 countries using the methods specified. In practice, it is often easier to select countries with large populations and feasible data collection infrastructure. An example of a 4-country selection could include the United States, Japan, Brazil, and Nigeria	Confirm survey feasibility, standardised protocol, translation quality, and local relevance of constructs	Low cost; risk of drawing broad conclusions from limited diversity
Team-driven collaboration (e.g., Everett et al., 2021)	Suitable when local context, cultural nuance, or face-to-face interaction is essential. Team composition can be based on principles of cultural coverage, not just convenience	Requires balancing standardisation (for comparability) with adaptation (for relevance). Important to ensure collaborators are full research partners, not just data collectors	Identify collaborators through initiatives like the Psychological Science Accelerator, ManyBabies, social media (e.g., Bluesky), or domain-specific networks	Pilot data from 1 to 3 sites; create shared protocol documents; pre-registration of methods and exclusion rules	Coordination-intensive; risk of protocol drift and inequity in recognition or leadership. Mitigated by transparent authorship plans and clear role definitions
Recruitment via social media (e.g., Thomson et al., 2018)	Ideal when researchers aim to emulate fieldwork across regions without physical presence. Works well for fast deployment of brief, engaging surveys	Instruments must be mobile-first, brief, and culturally neutral. Personalised feedback (e.g., relationship insights) can be integrated to boost engagement	Launch ad campaigns on platforms like TikTok or Instagram. Host surveys via Qualtrics or custom pages. Build feedback modules that generate participant-specific results	Monitor bounce rates, dropout, and time on task. Use A/B testing for ad variants. Track and validate back-end data logging	Medium cost for ad spend and hosting. Platform access varies by country; engagement may skew toward younger or older participants based on platform
Third-party participant panels (e.g., Awad et al., 2022)	Best suited for studies that require fast access to diverse national or regional samples. Ideal for testing hypotheses across multiple countries using pre-screened participants	Instrument length and complexity must match platform rules. Screening and quotas can help balance samples, but representation is limited to the vendor’s panel pool	Contact providers (e.g., BeSample, Cint, Lucid, Prolific, YouGov, Qualtrics Panels, Toluna). Define eligibility, quotas, and exclusion criteria. Request metadata access where possible	Note that “nationally representative” often excludes relevant traits beyond age and gender. Participants may complete multiple surveys in sequence, leading to carryover effects or fatigue. Use a single provider across countries where possible to ensure consistency	Medium to high cost. Some countries are underrepresented or unavailable. Limited control over recruitment pathways and metadata quality
Using existing historical or archival data (e.g., Chen et al., 2025; Islam et al., 2020; Jackson et al., 2023)	Best suited for studying naturally occurring variation, historical trends, or cultural content when primary data collection is infeasible	Research questions must align with what is already recorded. Analysis must adapt to pre-existing coding systems, measurement definitions, and units of observation	Sources are varied depending on the domain and research questions. Examples include the electronic Human Relations Area Files (eHRAF), COVID-19 policy trackers, Our World In Data, openICPSR, and Kaggle. May require merging datasets or applying natural language processing (NLP) to unstructured text	Validate construct alignment, source reliability, and completeness. Check translations, ethnographic coding, and sampling logic	Low financial cost, but high investment in data preparation. Risks include outdated variables, limited metadata, and non-comparable measures. Key constructs may be missing, requiring data combination or augmentation. Watch for definitional or algorithmic drift over time
Respondent-driven platform (e.g., Moral Machine) (see Box 1) (Awad et al., 2018)	Suitable for large-scale, global, organic reach via public interest in topics like tech, ethics, or identity, especially when demographic quotas are secondary to sample breadth	Discrete choice or preference designs must allow for scalable logic and analytic fallback plans (e.g., weighting, level restrictions). The interface must be intuitive, language-flexible, and mobile-first	Requires collaboration with designers and developers. Includes front-end interface design (e.g., React), gamified feedback, social sharing, and back-end infrastructure (e.g., Node.js, MongoDB Atlas, AWS)	Multiple testing phases: wireframe, prototype, alpha (logic and storage), beta (real-user feedback). Each non-trivial change requires full retesting due to dependencies	High resource demand and long timeline. Once launched, the platform must remain stable. Risk of low engagement without strong framing or media amplification

The observation that studies in social and behavioural sciences oversample from the Western world and that subjects from the Western world are unusual compared to other populations is not new (Arnett, 2008; Henrich et al., 2010a). The groundbreaking paper by Henrich et al. in 2010 (Henrich et al., 2010a, 2010b) provided a comprehensive overview of the problem, called for a broader sample of human participants from outside the Western world, and generated a lot of discussion (introducing WEIRD—Western, educated, industrialised, rich, democratic—as an acronym). Subsequently, many others wrote about this problem (Barrett, 2020; Cheon et al., 2020; Gurven & Lieberman, 2020). However, despite widespread agreement that this is a problem, little has been done to improve it.

The digital age has opened up opportunities to facilitate and support social science research with subjects from non-Western backgrounds. Here, I lay out the pathways researchers in these fields of psychological, behavioural, and social sciences can use to improve the representativeness and external validity of their findings.

## Digital pathways to diversify samples

The underrepresentation of non-Western participants is a serious problem. It means that our understanding of human cognition and behaviour is based on a very narrow sample of the population. This can lead to inaccurate conclusions and biased research. It is important to increase the representation of non-Western participants in psychological research so that we can develop a more accurate understanding of human psychology.

### Targeted site selection

The most parsimonious and least costly strategy would be to select 2–5 data collection sites that are culturally distant or broadly representative of the trait relevant to the research question. One way to do this is by using a theory-driven approach, such as the one used in a study (Rozin et al., 2016) comparing thinking patterns between East/South Asian cultures and Western culture drawing from an earlier work hypothesising “East—Right Hemisphere, West—Left Hemisphere” (Ornstein, 1972). A second approach is to use a cultural distance measure to choose locations. When literature is insufficient or conflicting, a more principled approach that synthesises both theoretical understanding and empirical data might be more useful. Several works have developed a distance scale measuring a (multi-dimensional) cultural distance between countries, and allocating them to culturally relevant classes or clusters (Inglehart & Welzel, 2005; Muthukrishna et al., 2020; Obradovich et al., 2020; Pick et al., 2022). While this parsimonious strategy provides an affordable option, it has several limitations: First, consistent behaviour across 2–3 culturally distant sites does not imply global generalisation. Small, non-representative samples or the omission of other cultural groups may fail to capture the full cultural spectrum. Second, differences in results across chosen countries do not necessarily indicate meaningful cultural variations, particularly if data collection protocols vary or findings conflict with theoretical or metric bases. Finally, it is advisable to avoid oversimplifying conclusions into West versus East (or West versus rest) dichotomies (Broesch et al., 2020; Masuda et al., 2020).

### Respondent-driven platform

Rather than taking a top-down strategy, one can also use an expansive (bottom-up) strategy, by trying to reach as many locations as they can. The advantages of this strategy are two-fold: firstly, reaching more countries (especially if diverse) can provide stronger evidence to support claims about hypothesised universals, and secondly, having a sufficient number of countries allows for analysing cultural and sociological predictors that can explain hypothesised variations. There are also two ways to go about this. The first one is respondent-driven; by posting the survey online and trying to reach out to as many respondents in different locations as possible. Since administering payment as incentive is more challenging in this case, one may want to rely on other forms of incentives like intrinsic motivation in the survey. One possible way is by employing gamification and online serious games (games with purposes beyond mere entertainment). An example of this is a research project I led called the Moral Machine (https://moralmachine.net), a website that gathers human perception of moral dilemmas faced by automated vehicles. The website eventually attracted over ten million participants from over 230 countries and territories, contributing to over 100 million decisions. It allowed us to study moral preferences across different dimensions simultaneously and across societies, showing that countries congregate in three large clusters (Awad et al., 2018). Capitalising on the success of the website, we ran another related study on it about three versions of the trolley problem (dilemma of sacrificing one to save many or not) across 42 countries (Awad et al., 2020). More recently, the use of serious games in social science research has become more mainstream (Long et al., 2023). When successful, this method can have multiple benefits such as their potential to scale, which itself has the advantage of a higher statistical power, better representation, and ability to study heterogeneity stemming from both demographics and experimental stimuli (Bryan et al., 2021). However, this method has its limitations. First, these projects involve considerable upfront costs—in terms of both monetary costs for development (and later maintenance) and time and deliberation for the painstaking planning and tweaking of the study design. Even then, the payoff in terms of reach is highly uncertain, requiring a flexible experimental design enough to make the most of the data if the website goes viral, but still be useful if only a small sample size is reached. Second, it has problems with representation (internet users, highly educated) and measurement (relying on short, pictorial prompts). Such problems may undermine attempts to reach non-Western samples. Indeed, a point that is particularly acute for digital strategies, but applies to other attempts to conduct cross-cultural research, is that collecting data from non-Western sites does not necessarily mean that the samples themselves will be non-Western. Highly educated participants from non-Western countries show similarity to Western participants (White & Muthukrishna, 2023), and so relying on such participants can understate or otherwise fail to represent cross-cultural differences. Further, the exclusive use of English language in places where it is a second language limits the reach to the more educated Westernised participants, in addition to biasing the cognitive constructs used and ignoring crucial variations in perception of fundamental concepts (Blasi et al., 2022). Therefore, it is essential to translate to other languages and collect data from nationally representative samples.

### Team-driven collaboration

The second way to use an expansive (bottom-up) strategy is team-driven: by recruiting collaborators (instead of participants) who form a diverse group of collection sites or are representative of culturally diverse locations (Coles et al., 2022). There are several examples for using this methodology, including consortiums like Psychological Science Accelerator (Jones et al., 2021) and ManyBabies (The ManyBabies Consortium et al., 2020), and the popularity of this method has increased during COVID-19 research, resulting in many cross-country big-team projects, including one I was involved in (Everett et al., 2021).

One advantage that this way has over the previous one is that it allows for more control over the representation of the samples. Another benefit is that it takes advantage of the familiarity of the co-authors of the local and cultural details in their own sites. However, it is important to ensure that collaborators are involved not only as data collection agents but as contributors to the conceptualisation, design, and authorship of the research. In many cases, structural incentives or standardisation pressures limit such involvement, but equitable collaboration should remain a central aim in team-based research (Puthillam et al., 2024).

Moreover, managing a large group of co-authors from different locations can be a daunting task, and it can lead to several problems if not properly managed. For one, reaching out to many researchers from several locations does not guarantee having a representative selection of sites (Schimmelpfennig et al., 2023) or even non-Westernised researchers (who are more likely than not to be Western-educated). Moreover, previous projects of this type used different protocols to collect data in different sites (e.g., some used face-to-face, others used an ad hoc online survey, while yet others used different third-party services). Such changes in protocols create additional confounders when the findings are different between countries.

### Recruitment via social media

Identifying ideal data collection sites and potential collaborators is merely a fraction of the journey; the essence of the mission lies in conducting the data-gathering process. Recently, digital tools opened the door for streamlining this process. Online “field studies”, for instance, can leverage the utility of social media platforms. One example is a study on relational mobility (Thomson et al., 2018), which successfully used Facebook ads and gamification to attract a total of 18,707 participants from 46 countries to fill surveys about their close friends and romantic partners in exchange of feedback about their respective relationships. These platforms can also support behavioural studies that observe user actions directly or implement randomised controlled interventions. However, such studies often require substantial technical setup or formal collaboration with the platform, and they raise important ethical considerations regarding participant consent and data use, such as the study by Facebook on emotional contagion (Kramer et al., 2014).

### Third-party participant panels

In the case of laboratory studies, third-party platforms can offer (moderately affordable) access to samples that approximate national representativeness of a diverse range of countries, often with quotas based on age, gender, race, or political affiliation. Providers such as YouGov, Qualtrics Panels, BeSample, Cint, Lucid, Toluna, and Dynata support screening for specific subpopulations and can offer nationally representative samples. However, “representativeness” usually refers only to a limited set of demographic features and does not ensure diversity across other relevant dimensions (e.g., income, religiosity, or even within-country regions). These platforms are best suited for survey experiments and hypothesis-testing studies requiring speed, structured demographic coverage, or cross-national comparison.

Researchers should be cautious of carryover effects, as participants often complete multiple surveys in quick succession, though in some cases, this remains an unavoidable limitation of panel-based research. Finally, to reduce risks of bias and inconsistency, it is advisable to use the same provider across countries, keep surveys concise, include attention checks, and—where possible—request regional stratification to support within-country analysis.

### Using existing historical or archival data

Capitalising on existing data provides another potent avenue for researchers to explore. This approach offers distinct advantages, primarily in the form of time and cost-efficiency, as it bypasses the need for new data collection. One such method is to examine revealed decisions or behaviour through natural experiments, as seen in numerous COVID-related papers; for example, the study of the association between physical distancing interventions and daily COVID-19 cases in 149 countries (Islam et al., 2020). On the other hand, stated preferences can be captured effectively using text analysis. For instance, using ethnographic data retrieved from the electronic Human Relations Area Files (eHRAF) (Ember, 2009), Jackson et al. (2023) used quantitative methods to analyse ethnographic text and sample 114 geographically and culturally diverse societies and used it to study supernatural explanations. However, archival data often lack direct measures of key constructs, requiring researchers to combine multiple sources or apply data augmentation techniques. For data collected over time, it is also important to watch for changes in coding practices, measurement definitions, or data-generating algorithms over time, which can introduce subtle forms of conceptual drift (Salganik, 2017).

Recent advances in natural language processing (NLP) offer new ways to extract behavioural signals from historical corpora. For instance, Chen et al. (2025) used a method called “cross-lingual questionnaire conversion” to match concepts from modern psychological surveys (in English) with semantically similar passages in classical Chinese writings. This approach makes it possible to trace psychological trends over centuries in culturally grounded ways (Chen et al., 2024).

## Strategic use of digital methods

Selecting the right approach to cross-cultural or globally distributed research depends not only on theoretical aims, but also on what is practically feasible given available resources, infrastructure, and team capacity. Each method comes with trade-offs: some offer scalability but little control, others provide rigour but require extensive coordination. To support researchers in making informed decisions, Table 1 summarises the six digital strategies mentioned above along five dimensions: strategic justification, study design considerations, operational setup, quality assurance, and resource and risk profile. These headings are meant to reflect the real-world constraints researchers must navigate. For the most resource-intensive but potentially high-reward strategy, respondent-driven public platforms, Box 1 provides a step-by-step implementation guide based on first-hand experience.

**Box 1** Building respondent-driven platforms (e.g., Moral Machine; Awad et al., 2018).

**Table:** 

**Strategic justification.** This approach is best suited for research questions that require large, organically diverse samples, often with global reach. It works especially well when the topic holds public relevance—such as morality, fairness, science, or emerging technologies. The design should appeal not only to research participants but also to those who discover the platform through media, curiosity, or social sharing. This method is not recommended when the study depends on tight experimental control, demographic quotas, or interviewer-guided tasks
**Study design considerations.** Scalability is key—both in the volume of responses and in the flexibility of design and analysis. A common approach is a discrete choice experimental design with conjoint analysis (Hainmueller et al., 2014), where participants evaluate profiles defined by combinations of attribute levels • Attribute combinations must be feasible: some combinations are not realistic; for example, when varying age and education levels, a profile of a candidate who is 8 years old with a PhD degree is implausible. These infeasible combinations must be ruled out in advance, which adds complexity to randomisation and analysis, but can be managed • To increase robustness, attribute levels can be prioritised by flagging a core subset as essential (e.g., appearing with higher frequency), while treating others as exploratory. This enables dual-track analysis: core-level models can be used when data are limited, while full designs can be explored when response volume is high. Non-uniform sampling requires adjustment during analysis via weighting or resampling • Interface design also matters. Visualisations should take priority over text. Where textual descriptions are necessary, they should appear only on demand (e.g., via hover or click), minimising cognitive load. Finally, incorporating playful or viral design elements—even those not central to the study—can boost shareability and engagement without compromising data quality
**Operational setup.** Platform flow should prioritise low friction and high engagement: minimise the number of clicks required before participants reach the first scenario or interactive task • **Landing page:** Present a concise pitch (e.g., *“Would you sacrifice one to save many? See what your choices reveal about you”*), clear consent language, and an optional language toggle • **Instructions:** Keep them short and visual. Avoid blocks of text; instead, use tooltips, examples, or progressive disclosure • **Task interface:** Present choices, ratings, or dilemmas using minimal UI—ideally without scrolling. Use icons or images where possible to reduce cognitive load • **Feedback:** Provide a personalised summary (e.g., *“You tend to prioritise fairness over loyalty”*), visualised and optionally downloadable. Encourage social media sharing with pre-filled text. Optionally offer another session • **Demographics and comments:** Always optional. These can appear just before feedback, immediately after, or in random order • **Gamification:** Essential for engagement. Include personality labels, scores, comparisons to others, or cumulative stats to help participants feel invested and curious about their results
**Quality assurance.** Generally, the development will have to go through several stages of testing **• Wireframe testing**: evaluate the basic layout, user flow, and content prioritisation **• Prototype testing**: check functionality, clarity of navigation between screens, and visual layout **• Alpha testing**: simulate user behaviour with synthetic data; confirm that randomisation, condition assignment, and data logging behave as intended **• Beta testing**: gather real users (e.g. team members, friends) to uncover edge cases, friction points, and comprehension failures Every non-trivial change (e.g., adding a new option or reordering stimuli) requires a full retesting of the platform due to dependencies between front-end logic and back-end storage
**Resource & risk profile** This method demands the most planning and infrastructure: • Working with experts in design (art and UX/UI) and web development early on to structure the experiment so that it scales and remains stable even under high traffic or inconsistent user input • Resist introducing new features or conditions after development begins: interdependencies often mean even small changes ripple across the system • Hosting via AWS (S3/CloudFront) and data storage via MongoDB Atlas are standard, but alternatives exist

Importantly, these strategies are not mutually exclusive. Combining them can improve both reach and representativeness. For example, in a study on the COVID-19 ventilator dilemma (Awad et al., 2022), we set the survey on the Moral Machine website allowing us to reach convenience samples from 20 countries. We augmented this sample with nationally representative samples via third-party panels from four countries that were chosen to represent three country clusters that we identified in another work on moral dilemmas (Awad et al., 2018). Combining three strategies allowed us to perform cross-country analysis as well as provide stronger evidence for universal patterns of preferences.

That said, digital methods have limitations. Firstly, they may only offer a restricted view of participants' lives, and they pose challenges in behavioural data collection without privacy-invasive tactics. Moreover, the financial requirements of some of these methods could further widen the resource gap between well-funded and budget-constrained researchers. Hence, these digital methods should complement, not replace, existing in-depth face-to-face approaches. Grant commissions and editors should encourage research using non-western samples, while being cognizant of the resource disparity across institutions and nations. They should also push researchers to redefine their target populations accordingly. Finally, there are situations when convenience Western samples are enough, such as when the findings are predominantly relevant to Western contexts, when the goal is to illustrate human deviations from theoretical models (Kahneman & Tversky, 2013), or when treatment effect heterogeneity across cultures is minimal (Coppock et al., 2018).

## Conclusion

Our understanding of the world, and of human cognition and behaviour in particular, is distorted by research based on the over-sampled Western populations. Skewed knowledge, amplified by the power of the internet, is consumed globally and often accepted as universal truths, thus creating a distorted representation of human cognition and behaviour. For instance, prominent psychological constructs such as the IQ tests, which heavily rely on individualism, dispositionality, and analytical thinking—all Western features (Henrich, 2020; Henrich & Muthukrishna, 2023)—have been primarily developed by Western researchers and validated on Western samples. Such measures have significantly influenced educational, organisational, and societal decision-making.

This amplification of distorted knowledge has recently entered a new era with the advent of large language models (LLMs), like OpenAI's GPT series (Brown et al., 2020), which present a new way of information dissemination. While these models have immense potential in democratising knowledge, they also risk perpetuating and amplifying the biases inherent in their training data. This risk has recently been documented with their performance in common behavioural tasks being shown to be more aligned with Western humans (Atari et al., 2023). Often, the information provided by these models masks the underlying sampling biases, making them less visible and potentially more pervasive.

Given this, as we navigate this new landscape of information and artificial intelligence, it is crucial to remember the importance of diverse and representative sampling in research. By actively seeking to go beyond Western-centric samples, we can strive to generate a more accurate, inclusive, and nuanced understanding of our world, a knowledge that is truly representative of the diverse tapestry of human experience.

## Data Availability

The data used to produce Fig. 1 come from two sources: (1) data from 2014 and 2017 are available in Tables 1 and 2 in Rad et al., 2018. Their raw data are available at the OSF link: https://osf.io/t2r87. (2) Raw data for 2023 were collected by the author of this article and are available at the OSF link: https://osf.io/kc8jt/?view_only=54e44062ea924e7a853d25ddde15457b.

## Code availability

Code is available at the OSF link: https://osf.io/kc8jt/?view_only=54e44062ea924e7a853d25ddde15457b.
